# Translesion synthesis DNA polymerase η exhibits a specific RNA extension activity and a transcription-associated function

**DOI:** 10.1038/s41598-017-12915-1

**Published:** 2017-10-12

**Authors:** Vamsi K. Gali, Eva Balint, Nataliia Serbyn, Orsolya Frittmann, Francoise Stutz, Ildiko Unk

**Affiliations:** 1 The Institute of Genetics, Biological Research Centre, Hungarian Academy of Sciences, Szeged, H-6726 Hungary; 2 Department of Cell Biology, iGE3, University of Geneva, 1211 Geneva, Switzerland; 3 Present Address: Institute of Medical Sciences Foresterhill, University of Aberdeen, Aberdeen, United Kingdom

## Abstract

Polymerase eta (Polη) is a low fidelity translesion synthesis DNA polymerase that rescues damage-stalled replication by inserting deoxy-ribonucleotides opposite DNA damage sites resulting in error-free or mutagenic damage bypass. In this study we identify a new specific RNA extension activity of Polη of *Saccharomyces cerevisiae*. We show that Polη is able to extend RNA primers in the presence of ribonucleotides (rNTPs), and that these reactions are an order of magnitude more efficient than the misinsertion of rNTPs into DNA. Moreover, during RNA extension Polη performs error-free bypass of the 8-oxoguanine and thymine dimer DNA lesions, though with a 10^3^ and 10^2^–fold lower efficiency, respectively, than it synthesizes opposite undamaged nucleotides. Furthermore, *in vivo* experiments demonstrate that the transcription of several genes is affected by the lack of Polη, and that Polη is enriched over actively transcribed regions. Moreover, inactivation of its polymerase activity causes similar transcription inhibition as the absence of Polη. In summary, these results suggest that the new RNA synthetic activity of Polη can have *in vivo* relevance.

## Introduction

When DNA replication is blocked by DNA lesions, DNA damage tolerance (DDT) mechanisms are activated that can sustain replication on damaged templates without removing the damage. One mechanism of DDT is translesion synthesis (TLS), where specialized DNA polymerases synthesize across the damage and/or extend from it^1^. TLS polymerases can be found in all three domains of life. They display lowered selectivity and fidelity compared to replicative polymerases due to their large active center and to their lack of a proofreading activity. Their non-selective active center can accommodate damaged and modified bases enabling them to perform TLS. Damage bypass can be error-free or error-prone depending on whether the correct or an incorrect nucleotide is inserted opposite a lesion. Error-free bypass contributes to genomic stability, whereas error-prone damage bypass increases instability by causing mutagenesis. The *RAD30*-encoded TLS DNA polymerase Polη of *Saccharomyces cerevisiae* can bypass several DNA lesions in a faithful or mutagenic manner. It stands out among other polymerases by its unique ability to efficiently and accurately bypass cyclobutane pyrimidine dimers (CPDs), the most frequent UV-induced DNA lesions^2^. It can do so because its active center can accommodate both nucleotides of the dimer^3^. Polη can also bypass efficiently and error-freely 8-oxoguanine (8-oxoG), one of the most abundant spontaneous oxidative lesions, whereas replicative DNA polymerases carry out mostly error-prone bypass of this damage^4^. Though Polη is highly error-prone on non-damaged DNA, its inactivation in yeast cells and in mouse cell lines increases the UV-induced mutation rate^5–7^, while in humans it causes a cancer-prone syndrome, the variant form of xeroderma pigmentosum^8,9^. These findings indicate that the main *in vivo* function of Polη is non-mutagenic and its activity is mostly restricted to damage sites. The distributive mode of DNA synthesis by Polη, that dissociates from DNA after inserting only a few nucleotides, probably also serves to confine its activity^10^. Polη can get access to the stalled replication fork through its interaction with proliferating cell nuclear antigen (PCNA), the processivity clamp of the replicative DNA polymerases^11,12^. The interaction with PCNA is essential for the *in vivo* function of Polη, as mutations disrupting this interaction cause the same phenotype in yeast as the complete lack of Polη^11^.

DNA polymerases use deoxy-ribonucleotides (dNTPs) when synthesizing DNA, despite the much higher cellular concentration of ribonucleotides (rNTPs). It was discovered that a specific amino acid, called the “steric gate”, is responsible for exclusion of rNTPs from the active site of DNA polymerases^13–15^. However, the exclusion is not complete and even the major replicative DNA polymerases have been shown to insert rNTPs during DNA synthesis with varying low frequency^16–20^. The presence of ribonucleotides in the genome is destabilizing^21^, and is counteracted by ribonucleotide excision repair that efficiently removes ribonucleotides from the genomic DNA^22^.

In this study we show that Polη is inefficient in inserting rNTPs during DNA synthesis, but unexpectedly, it has the specific activity to extend RNA strands with ribonucleotides. Moreover, Polη can mediate RNA TLS during RNA extension with the same fidelity as it does during DNA synthesis, although with very low efficiencies. Polη can also insert dNTPs into RNA, and it does so with similar efficiencies as with rNTPs at nucleotide concentrations estimating the *in vivo* conditions. Moreover, damage bypass by Polη is more efficient with dNTPs during RNA extension. Nevertheless, Polη is required for the efficient transcription of several genes *in vivo* and is physically associated with the open reading frame of the actively transcribed *GAL1* gene. Furthermore, we demonstrate that the polymerase activity of Polη is required for its transcription-associated function *in vivo*. Based on our findings we propose a role for the discovered new RNA synthetic activity of Polη during transcription.

## Results

### Polη has the specific activity to extend RNA strands with ribonucleotides

**Figure 1:**
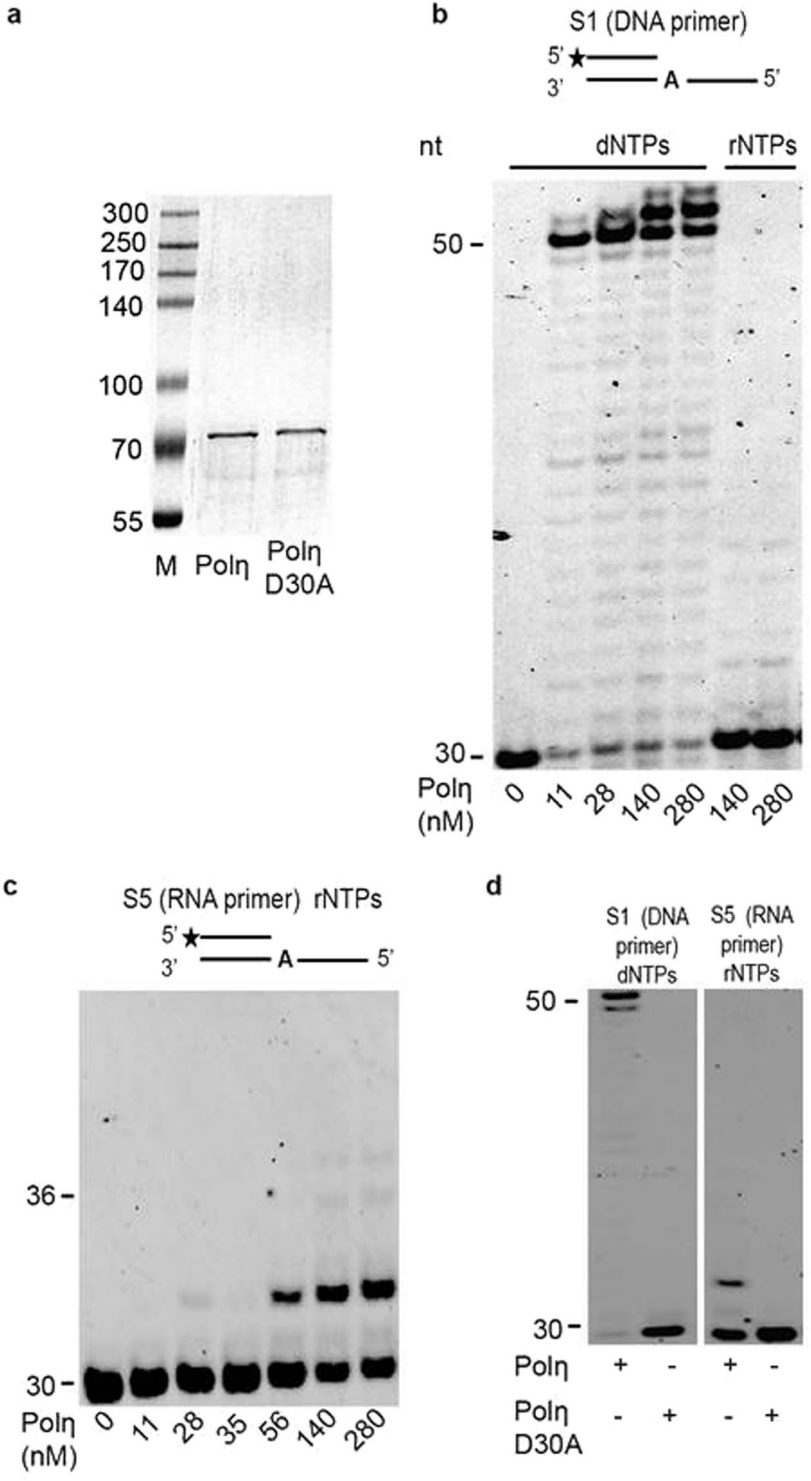
Polη can catalyze DNA and RNA extension with rNTPs. (**a**) Purity of recombinant Polη and Polη D30A. 200 ng of each protein was analyzed on 8% denaturing SDS-polyacrylamide gel. Molecular mass standards are shown on the left. (**b**) Polη can perform DNA primer extension with dNTPs and rNTPs. Reactions were carried out using 24 nM template and increasing concentrations of Polη, as indicated at the bottom, in the presence of 100 μM dNTP or rNTP for 5 min. (**c**) Polη can catalyze RNA primer extension with ribonucleotides. Reactions contained 16 nM template and 100 µM rNTP and were incubated for 10 min. (**d**) Polη D30A is defective in both DNA and RNA primer extensions. Reactions were incubated with wild-type or mutant Polη (140 nM), in the presence ﻿of 100 µM of either dNTPs (left panel) or rNTPs (right panel) for 10 min. The structures of the substrates are shown at the top of each panel. Asterisks mark the 5′ Cy3 labeled ends. See Fig. S1 for full-length images.

We examined whether Polη could use rNTPs when synthesizing DNA, by performing *in vitro* primer extension assays in the presence of purified recombinant Polη (Fig. 1a). The activity of Polη was confirmed in control DNA extension reactions in the presence of dNTPs (Fig. 1b). When rNTPs were added to the reactions instead of dNTPs, Polη was still able to extend the DNA primer using ribonucleotides (Fig. 1b). Although this extension was very inefficient and required high enzyme concentrations, Polη could synthesize a ribonucleotide chain on the DNA primer using rNTPs, as indicated by the appearance of lower mobility bands on the gel. Thus, the terminal ribonucleotide did not inhibit further synthesis and Polη was able to extend not only a terminal deoxy-ribonucleotide, but also a terminal ribonucleotide containing primer. This prompted us to investigate the ribonucleotide chain extension ability of Polη in reactions containing a DNA template hybridized with an RNA primer in the presence of rNTPs. Importantly, these experiments demonstrated that Polη was able to extend an RNA primer with rNTPs and to catalyze the formation of a polyribonucleotide chain (Fig. 1c). The absence of any polymerase activity when using the catalytically inactive Polη D30A mutant in these assays confirmed that both the DNA and RNA synthetic activities are intrinsic to Polη (Fig. 1d). Notably, the extension of a primer with dNTPs or rNTPs results in slightly different electrophoretic mobility; it can therefore be ruled out that the observed activity results from contamination of the rNTPs by dNTPs (Fig. 1b last two rows, and Fig. S2).

**Figure 2:**
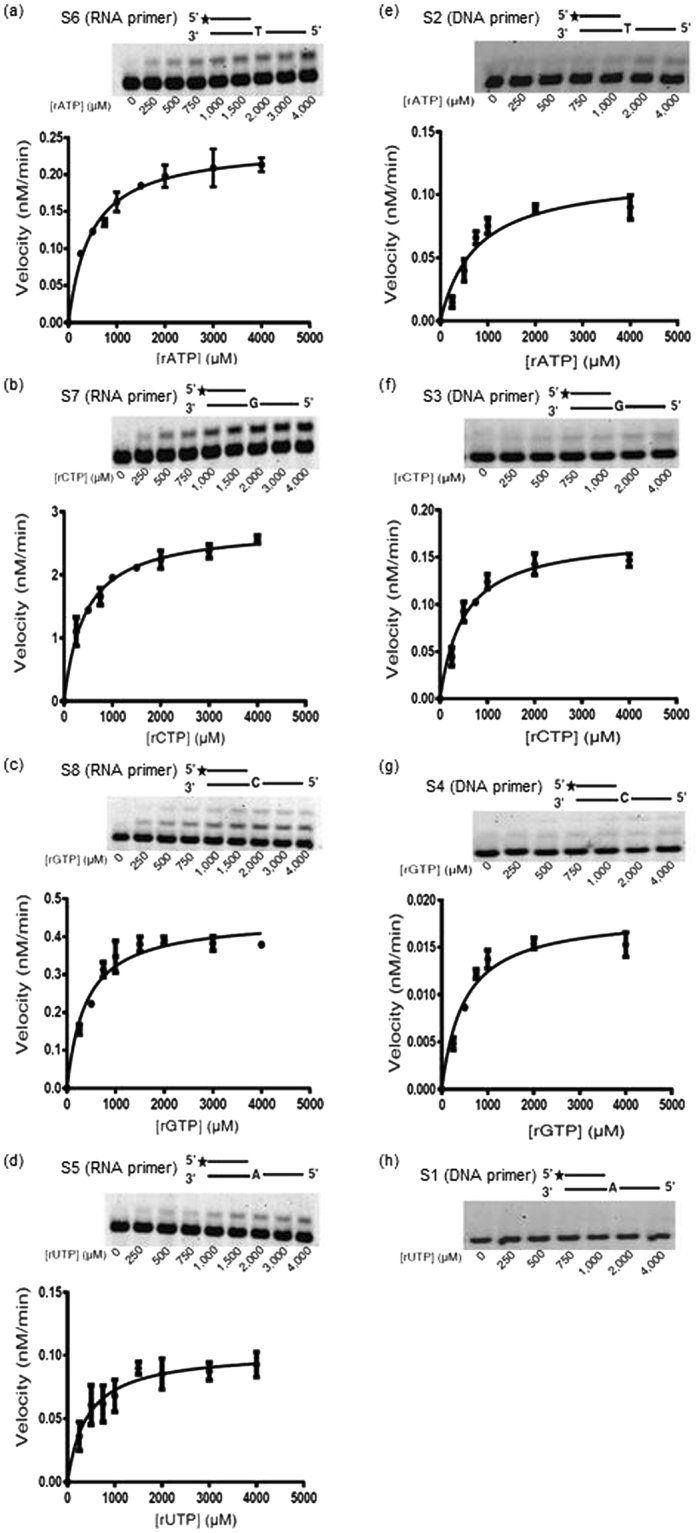
Steady-state kinetic analysis of RNA and DNA primer extensions with rNTPs by Polη. RNA primer extension with (**a**) rATP, (**b**) rCTP, (**c**) rGTP, (**d**) rUTP is shown on the left. DNA primer extension with (**e**) rATP, (**f**) rCTP, (**g**) rGTP, (**h**) rUTP is shown on the right. Polη (1 nM) was incubated with 20 nM of the indicated templates in the presence of increasing concentrations of the single incoming rNTP, as shown under the gel pictures. The quenched samples were analyzed by denaturing polyacrylamide gel electrophoresis, and quantified as described (see Materials and Methods). For each rNTP the rate of incorporation is plotted as a function of rNTP concentrations. The data were fit to the Michaelis-Menten equation (see Materials and Methods). In panel (h) incorporation of rUTP was so weak that it could not be quantified. See Fig. S3 for full-length images.

Polη seemed rather inefficient in RNA extension with rNTPs as opposed to DNA extension with dNTPs. For example, at 11 nM enzyme concentration Polη extended nearly all the DNA primers in the reaction with dNTPs, whereas almost no insertion of rNTPs into RNA could be observed at the same enzyme concentration (compare Fig. 1b and c). However, the applied 100 µM dNTP concentration was much higher than the intracellular dNTP level that ranges from 12–30 µM, and vice versa, the applied 100 µM rNTP concentration was much lower than the intracellular rNTP level of 0.5–3 mM^16^. To clarify whether the RNA extension ability of Polη reflected a specific activity or was the result of misinsertion, we performed steady-state kinetic analysis where we compared the efficiency of rNTP incorporation by Polη into RNA versus DNA. Remarkably, Polη extended RNA primers with rNTPs an order of magnitude more efficiently than DNA primers, except in the case of rATP (Fig. 2 compare **a** to **e**, **b** to **f**, **c** to **g**, and **d** to **h**; Tables 1 and 2). For example, Polη incorporated rGTP into RNA ~30, and rCTP ~20 times more efficiently than into DNA, whereas rUTP incorporation into DNA was so weak that it was not measurable (Fig. 2h). Significantly, the *K*_*m*_ values for RNA extension with single rNTPs were in the range of the intracellular concentrations of rNTPs suggesting that the activity might have an *in vivo* relevance. In summary, these results show that Polη recognizes RNA as its substrate and that rNTP incorporation into RNA is specific and not merely misincorporation due to the not-so-stringent active center of Polη.

**Table 2:** Kinetic parameters of rNTP incorporation into DNA and RNA by Polη.

Primer	Insertion opposite	Incoming riboucleotide	Kcat (min-1)	Km (µM)	Kcat/Km	Relative efficiency^a^
RNA	T	ATP	0.2394 ± 0.0065	466.4 ± 47.29	5.13E-04	3.34
RNA	G	CTP	2.758 ± 0.06217	438.3 ± 37.52	62.9E-04	18.26
RNA	C	GTP	0.4487 ± 0.01485	393.7 ± 52.04	11.4E-04	30.24
RNA	A	UTP	0.1032 ± 0.005715	423.3 ± 90.45	2.43E-04	n.d
DNA	T	ATP	0.1163 ± 0.009014	757.6 ± 160	1.53E-04	
DNA	G	CTP	0.1733 ± 0.007439	503.1 ± 68.83	3.44E-04	
DNA	C	GTP	0.01851 ± 0.000891	491.2 ± 76.14	0.37E-04	
DNA	A	UTP	—	—	—	

Values were obtained from results shown in Fig. 2, and represent the mean and standard error of three experiments. Kinetic parameters were calculated as described in “Materials and Methods”. n.d, not determined (no insertion of UTP into DNA could be detected). Relative efficiency was calculated using the following equation: *f*_*ext*_ = (*k*_*cat*_ */K*_*m*_)_RNA_/(*k*_*cat*_ */K*_*m*_)_DNA_.

### Polη can extend RNA with deoxy-ribonucleotides

**Figure 3:**
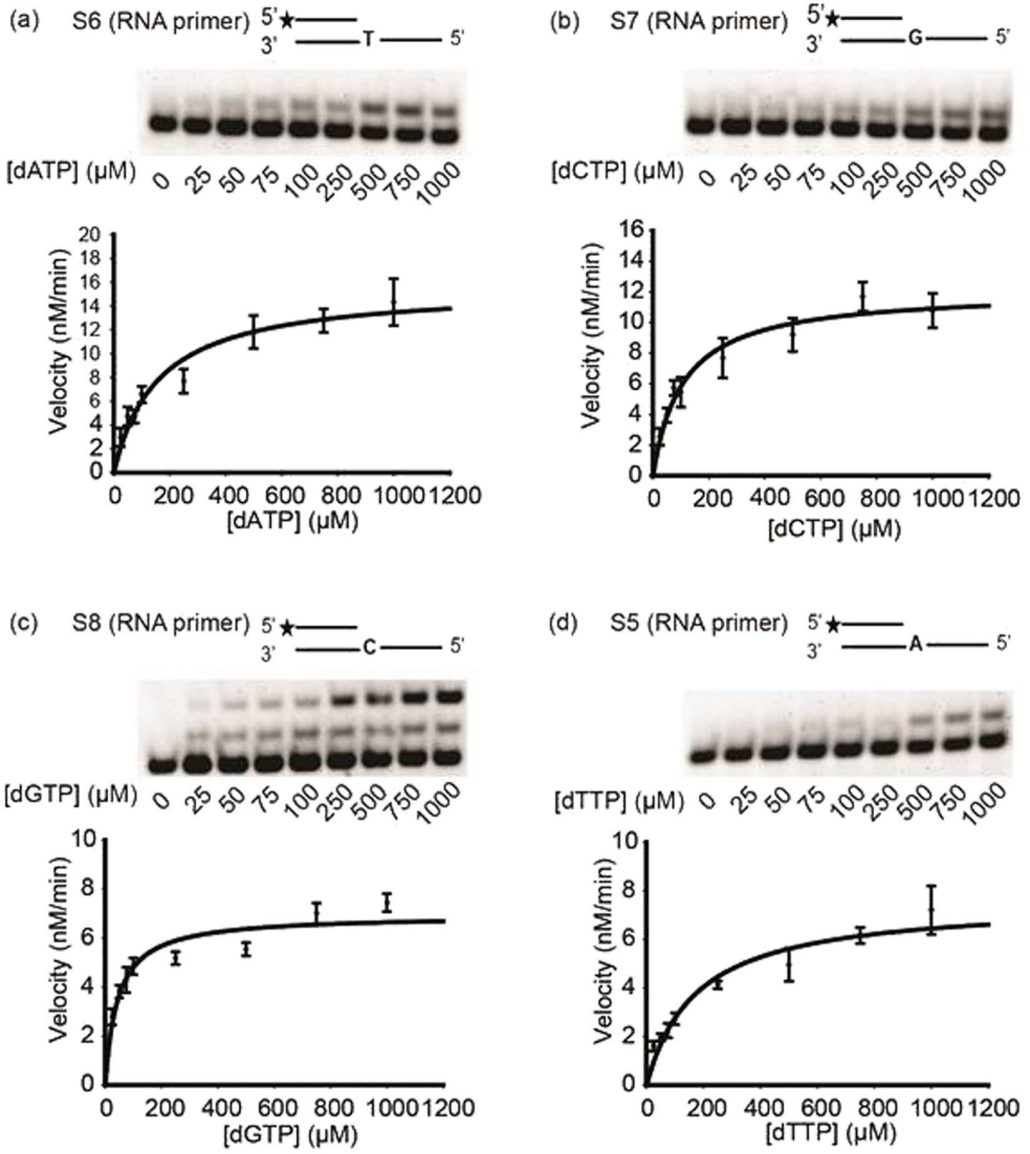
Steady-state kinetic analysis of RNA extension with dNTPs by Polη. Gel pictures of RNA primer extensions with (**a**) dATP, (**b**) dCTP, (**c**) dGTP, (**d**) dTTP are shown at the top of each panel. Polη (1 nM) was incubated with 24 nM of the indicated templates in the presence of increasing concentrations of the single incoming dNTP, as shown under the gel pictures. The quenched samples were analyzed by denaturing polyacrylamide gel electrophoresis, and quantified as described (see Materials and Methods). For each dNTP the rate of incorporation is plotted as a function of dNTP concentrations. The data were fit to the Michaelis-Menten equation (see Materials and Methods). See Fig. S4 for full-length images.

Next we investigated whether Polη selectively inserted rNTPs during RNA synthesis, or whether dNTP misinsertion could also occur. For this reason we applied single dNTPs in the RNA primer extension reactions and determined the kinetic parameters of the reactions (Fig. 3 and Table 3). As our steady-state kinetic assays showed, the K_cat_/K_m_ values for dNTP insertions were much higher compared to rNTP insertions indicating that dNTP insertions were more effective. However, when we took into consideration the big difference between the *in vivo* concentrations of dNTPs and rNTPs, the relative frequencies were around 1 (Table 3) meaning that at physiological dNTP and rNTP concentrations Polη inserts dNTPs and rNTPs into RNA with similar efficiencies.

**Table 3:** Kinetic parameters of dNTP or rNTP incorporation into RNA by Polη.

Insertion opposite	Incoming nucleotide	k_cat_ (min^−1^)	K_m_ (µM)	k_cat_/K_m_	nucleotide concentration^a^ (µM)	Relative frequency
T	dATP	15.607 ± 1.227	158.94 ± 39.16	98.19 E-03	16	1.02
T	rATP	0.2394 ± 0.0065	466.4 ± 47.29	0.51 E-03	3000	
G	dCTP	12.085 ± 0.786	107.7 ± 24.7	112.20 E-03	14	0.52
G	rCTP	2.758 ± 0.062	438.3 ± 37.52	6.29 E-03	500	
C	dGTP	6.9332 ± 0.2768	44.35 ± 7.92	156.31 E-03	12	2.35
C	rGTP	0.4487 ± 0.0149	393.7 ± 52.04	1.14 E-03	700	
A	dTTP	7.5753 ± 0.5505	174.49 ± 38.01	43.41 E-03	30	3.14
A	rUTP	0.1032 ± 0.0057	423.3 ± 90.45	0.24 E-03	1700	

^a^ *In vivo* dNTP and rNTP concentrations are according to ref.^14^.

Values were obtained from results shown in Figs 2 and 3, and represent the mean and standard error of at least three experiments. Relative frequency of incorporation was calculated using the following equation: *f*_*rel*_ = (k_cat1_ */*K_m1_) * [dNTP]**/**(k_cat2_/K_m2_) * [rNTP], where k_cat1_/K_m1_ is the value for dNTP incorporation and k_cat2_/K_m2_ is the one for rNTP incorporation.

### Polη can perform error-free bypass of an 8-oxoG and a TT dimer during RNA extension

**Figure 4:**
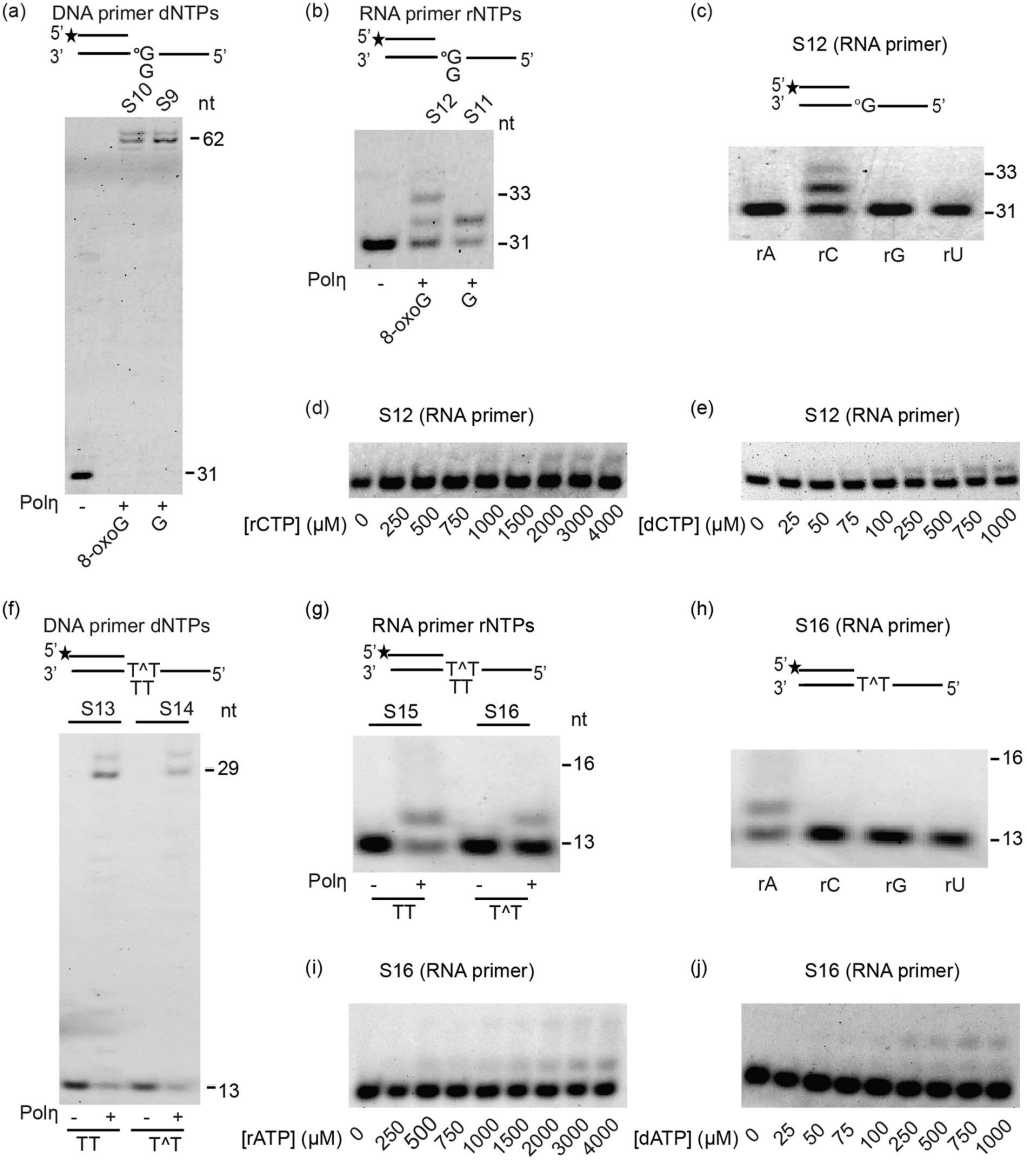
Polη can carry out error-free bypass of 8-oxoG and TT dimer during RNA extension. (**a**) Polη (56 nM) can synthesize through a G, and an 8-oxoG containing template (8 nM both) during DNA extension with dNTPs (100 µM) and (**b**) also during RNA extension with rNTPs (100 µM) (**c**) Polη (10 nM) inserts only the correct C (4 mM of each rNTP) opposite 8-oxoG (8 nM). (**d**) Kinetic assay for rCTP and (**e**) dCTP insertion opposite 8-oxoG. (**f**) Polη (18 nM) can synthesize through an undamaged TT and a TT dimer containing template (16 nM both) during DNA extension with dNTPs (100 µM), as well as (**g**) during RNA extension with rNTPs (1 mM). (**h**) Polη (18 nM) inserts only the correct A (4 mM of each rNTP) opposite a TT dimer (16 nM). (**i**) Kinetic assay of rATP and (**j**) dATP insertion opposite a TT dimer. In reactions shown in (**d,e**) 1 nM Polη was incubated with 8 nM template. In (**i,j**) reactions contained 1 nM Polη and 16 nM template. Conditions for the kinetic assays of (**d,e,i,j**) are detailed in Materials and Methods. The positions of the normal G or 8-oxoG (°G), and the two Ts or the TT dimer (TΛT) in the substrates are indicated Asterisks mark the 5′ Cy3 labeled ends. See Fig. S5 for full-length images.

The main identified cellular function of Polη is to promote DNA replication through DNA damages by inserting dNTPs opposite to damage sites. To test whether it exhibits similar activity during RNA extension, we examined Polη damage bypass ability *in vitro* using an 8-oxoG, or a TT dimer containing oligonucleotide. We chose these DNA lesions because Polη was already shown to bypass them efficiently and in an error free manner during DNA synthesis^2,4^. We confirmed that Polη can bypass these DNA lesions during DNA synthesis with dNTPs (Fig. 4a and f). Furthermore, Polη was able to extend the RNA primer opposite an 8-oxoG (Fig. 4b) and a TT dimer (Fig. 4g) with rNTPs. More importantly, even when high 4 mM single rNTP concentrations were included in the reactions, it inserted only CTP opposite 8-oxoG (Fig. 4c) and only ATP opposite the TT dimer (Fig. 4h). The result showing 2 rNTP insertions opposite 8-oxoG, but only 1 opposite the undamaged C (Fig. 4b) is in good agreement with the observation that Polη is more processive on damaged DNA^23^. The weak intensity of the bands in Fig. 4g corresponding to multiple insertions is probably due to the applied lower enzyme/template ratio. In summary, these results show that Polη bypasses 8-oxoG and TT dimer in an error-free manner during RNA synthesis by inserting only the corresponding correct rNTPs opposite the lesions.

### Polη can bypass DNA damage with dNTPs during RNA extension

Next we checked the efficiencies of the bypass reactions by kinetic analysis applying the single correct incoming rNTPs (Fig. 4d and i, and Fig. S6). For both 8-oxoG and TT dimer bypass we obtained very low K_cat_/K_m_ values, 1000 times and 100 times lower numbers than measured on undamaged templates, respectively, reflecting the inefficient nature of the reactions (Table 4). Repeating the experiments using the corresponding single dNTPs instead of rNTPs revealed that lesion bypass during RNA synthesis is more efficient when Polη inserts dNTPs (Fig. 4e and j, Fig. S6 and Table 4). As Table 4 shows, even at intracellular nucleotide concentrations both 8-oxoG and TT dimer bypass are ~19 times more efficient with dNTPs compared to rNTPs. In summary, these experiments indicate that although Polη is able to carry out error-free DNA lesion bypass during RNA extension using rNTPs, it preferentially inserts dNTPs opposite these lesions.

**Table 4:** Kinetic parameters of dNTP or rNTP incorporation by Polη into RNA opposite DNA damages.

Insertion opposite	Incoming nucleotide	k_cat_ (min^−1^)	K_m_ (µM)	k_cat_/K_m_	nucleotide concentration^a^ (µM)	Relative frequency
oxoG	dCTP	1.7018 ± 0.1177	69.85 ± 18.53	24.36 E-03	14	19.4
oxoG	rCTP	0.03424 ± 0.00358	973.97 ± 270.44	3.53 E-06	500	
TT dimer	dATP	4.9105 ± 0.5331	287.12 ± 83.85	17.10 E-03	16	18.6
TT dimer	rATP	0.00831 ± 0.00106	1677.9 ± 444.8	4.95 E-06	3000	

^a^ *In vivo* dNTP and rNTP concentrations are according to ref.^14^.

Values were obtained from results shown in Fig. 4, and represent the mean and standard error of three experiments. Relative frequency was calculated using the formula as for Table 3.

### *rad30Δ* cells are sensitive to transcription inhibitors

**Figure 5:**
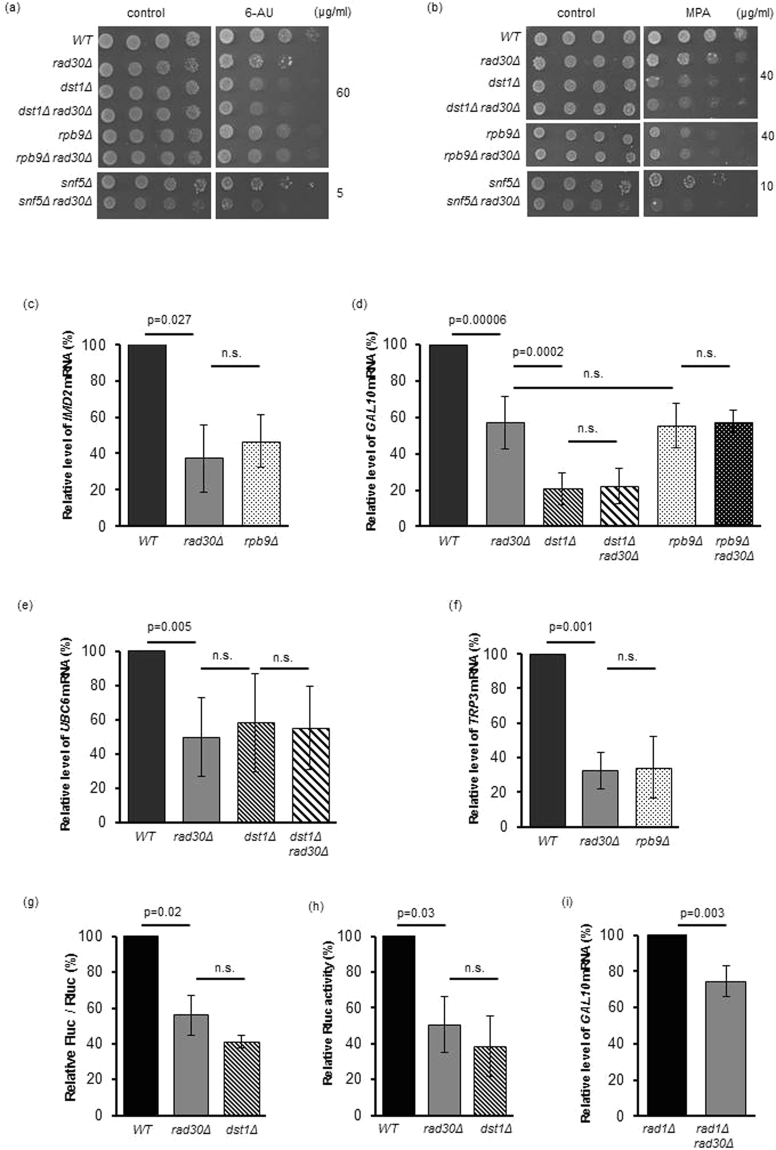
The absence of *RAD30* affects transcription. (**a**) *rad30Δ* strains exhibit sensitivity to the elongation inhibitor 6-azauracil (6-AU) and (**b**) to mycophenolic acid (MPA). Ten fold serial dilutions of the strains with the indicated genotypes were spotted on media containing the indicated concentration of the appropriate drugs. (**c**) 6-AU induced mRNA level of the *IMD2* gene and (**d**) galactose induced mRNA level of the *GAL10* gene in the presence of MPA decreased in the *rad30Δ* strain, as measured by RT-qPCR. (**e**) Constitutive expressions of the *UBC6* and (**f**) *TRP3* genes in the presense of 6-AU are decreased in the absence of *RAD30*, as measured by RT-qPCR. (**g**) Firefly luciferase activity expressed from the induced *GAL1* promoter is lowered in *rad30Δ* cells. (**h**) Renilla luciferase activity, driven from the constitutive *GPD* promoter, is diminished in the absence of *RAD30*. (**i**) Deletion of *RAD30* causes transcriptional defect even in G1-arrested, NER defective cells, as measured by RT-qPCR. Panels (c–f and i) show relative mRNA levels measured by RT-qPCR normalized to *SED1* mRNA. In panels (**g,h**) relative enzyme activities are presented. In panels (**c–i**) the values obtained for the wild-type strain were set to 100% and the values obtained with the deletion strains are shown relative to that. Data are presented as mean ± SD of at least 3 experiments. p values are indicated, ns: no statistical difference.

To investigate whether the newly discovered RNA synthetic activity of Polη can have functional significance, we asked whether Polη could be linked to transcription. To address this question, we first investigated the sensitivity of yeast *rad30* deletion strains to the widely used transcription inhibitor 6-azauracil (6-AU). 6-AU depletes the cellular levels of the RNA precursors UTP and GTP by inhibiting IMP dehydrogenase^24^. Consequently, transcription elongation becomes susceptible to perturbations and as a result, many elongation mutants were shown to exhibit sensitivity to the drug^25–27^. Surprisingly, *rad30Δ* cells showed marked sensitivity to 6-AU compared to wild-type cells (Fig. 5a). This prompted us to investigate the relationship of *RAD30* to genes involved in transcription. We examined *DST1* coding for the canonical elongation factor TFIIS, the elongation factor gene *RPB9* coding for a small subunit of RNAPII, and *SNF5* encoding a chromatin remodeler involved in transcriptional activation^28–30^. Whereas additional deletion of *RAD30* did not change the 6-AU sensitivities of the *dst1Δ* and *rpb9Δ* elongation factor mutant strains, it further sensitized the *snf5Δ* strain (Fig. 5a). Similar results were obtained using mycophenolic acid (MPA), another inhibitor of IMP dehydrogenase (Fig. 5b)^31^, suggesting that the absence of Polη might cause a defect in transcription.

### The lack of *RAD30* affects inducible and constitutive gene expression

Sensitivity to 6-AU and MPA can be indicative of transcriptional defects, however, deletion of some genes involved in other cellular processes also confer sensitivity to these drugs^32^. On the other hand, reduced transcriptional induction of the *IMD2* gene, encoding IMP dehydrogenase, by 6-AU or MPA is characteristic of genuine transcriptional mutants^26^. To define whether the observed 6-AU sensitivity of *rad30Δ* cells actually reflected impairment of transcription, we first examined the induced synthesis of the *IMD2* mRNA in the presence of 6-AU by reverse transcription followed by real time quantitative PCR (RT-qPCR). Indeed, 60% reduction in transcription, as monitored by *IMD2* induction, was observed in *rad30Δ* cells suggesting that *RAD30* contributes to efficient gene expression (Fig. 5c). Investigation of two other commonly examined loci the galactose inducible *GAL10* and *GAL1* genes^33,34^, strengthened these results. As shown in Fig. 5d and Fig. S7, a ~40% decrease in the *GAL10* and *GAL1* mRNA levels could be detected in *rad30Δ* cells compared to the wild-type strain. The *dst1Δ* and *dst1Δ rad30Δ*, as well as the *rpb9Δ* and *rpb9Δ rad30Δ* mutants exhibited comparable levels of *GAL* gene expression, consistent with the 6-AU sensitivities of these strains. Next we tested whether constitutive transcription was also affected in *rad30Δ* by investigating the *UBC6* and *TRP3* genes whose mRNA levels were shown to be stable in wild-type cells^35,36^. We found that the expression of these genes was reduced by ~50–70% in *rad30Δ* cells compared to wild-type cells (Fig. 5e and f). Transcription levels in *rad30Δ* cells were also monitored by luciferase reporter assays. We measured the activity of the firefly luciferase driven from the induced *GAL1* promoter and observed a ~40% decrease in the *rad30Δ* compared to the wild-type strain, whereas the decrease was ~60% in the *dst1Δ* mutant (Fig. 5g). Similarly, the activity of the renilla luciferase expressed from the strong, constitutive glyceraldehyde-3-phosphate dehydrogenase promoter decreased to ~50% of the wild-type level in *rad30Δ* cells (Fig. 5h). These results support the view that deletion of *RAD30* influences transcription causing a marked decrease in the mRNA levels of different genes.

### *rad30Δ* cells exhibit transcriptional defect even when DNA synthesis is inhibited

Our next aim was to define whether the observed transcriptional defect of *rad30Δ* cells could originate from the role of Polη in DNA synthesis. We surmised that in the absence of Polη, replication complexes could stall more frequently and for longer times resulting in the block of transcription; alternatively, single-stranded gaps generated by NER could inhibit transcription if Polη was involved in the gap-filling step. This latter assumption takes into consideration the findings that the TLS DNA polymerases, mouse Polκ and yeast Polζ function in NER as well^37,38^. Also, in *Escherichia coli* the TLS DNA polymerase DinB was found to interact with the transcription elongation factor NusA^39^. It was suggested that NusA recruits DinB to transcription complexes stalled at single-stranded gaps generated by NER, where it participates in gap-filling. To investigate these possibilities, we examined a *rad1Δ rad30Δ* double mutant strain arrested in the G1 phase of the cell cycle. Under these conditions NER is inactive due to the lack of the Rad1 endonuclease essential for NER, and replication is inhibited by cell cycle arrest. Importantly, even under these conditions, *rad30Δ* cells displayed defects in *GAL10* and *GAL1* gene transcription similarly to the previous experiments indicating that the observed effect on transcription was independent of the role of Polη in replication and in repair synthesis (Fig. 5i and Fig. S7). In addition, since transcription-coupled NER is non-functional in the absence of Rad1, the transcriptional impairment of *rad30Δ* cells could not stem from a possible involvement of Polη in this process.

### Deletion of *RAD30* affects transcription elongation *in vivo*

**Figure 6:**
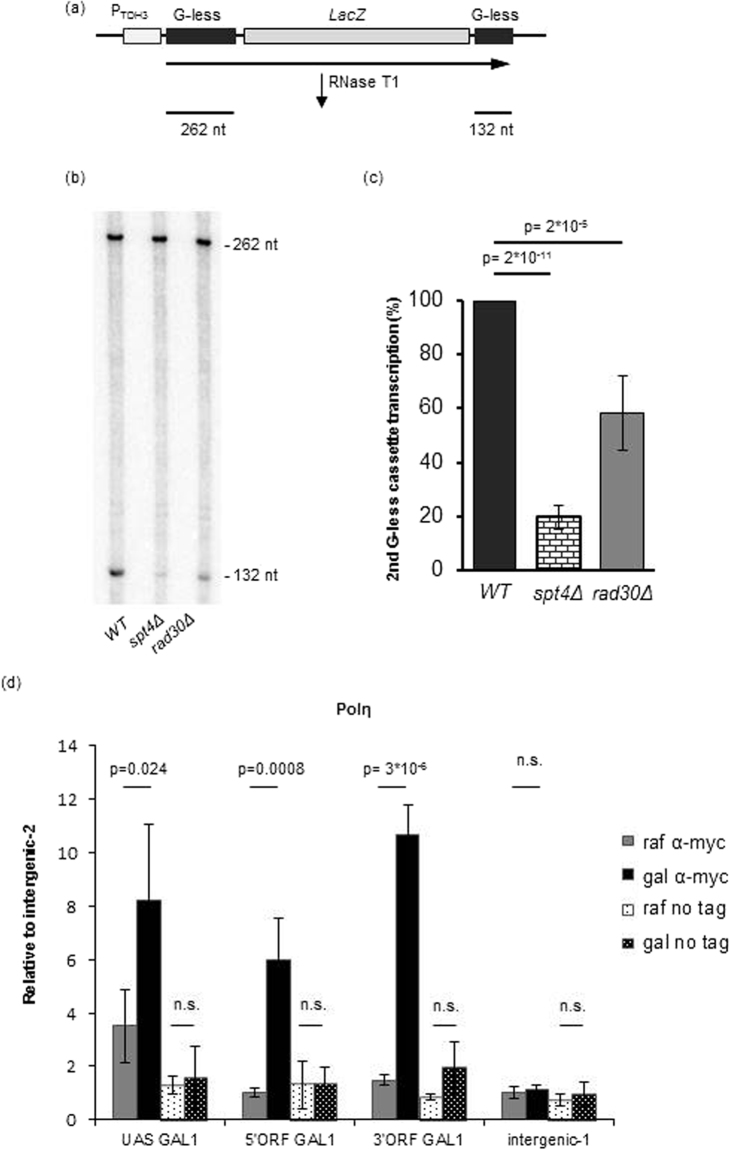
Polη affects elongation *in vivo* and is enriched over the active *GAL1* gene (**a**) Schematic drawing of the G-less based run-on (GLRO) assay. The sizes of the two G-less cassettes are shown. (**b**) GLRO analysis was performed with the indicated strains transformed with the GLRO-long plasmid. A representative gel picture is shown. See Fig. S8 for the full-length image. (**c**) Quantification of the results of four independent GLRO experiments. For each sample, the ratio of total counts incorporated into the distal versus the proximal G-less cassettes was normalized to the ratio in the wild-type strain, which was set to 100%. (**d**) Occupancy of Polη on the UAS, 5′ORF, 3′ORF of the *GAL1* gene and on two independent intergenic regions in uninduced (raf) and induced (gal) conditions was measured by chromatin immunoprecipitation (ChIP) using anti-Myc antibody in a strain arrested in G1, and expressing C-terminally Myc-tagged Polη. As control, ChIP was also performed with an untagged strain (no tag). Percentage of input at the indicated regions was normalized to intergenic region 2 on chromosome IV. Experiments were repeated at least 3 times. Mean and standard deviations are indicated, p-values were calculated by 2-tailed t-test, n.s.: no statistical difference.

To further corroborate the connection between *RAD30* and transcription, we examined transcription elongation by employing the G-less-based run-on (GLRO) method developed for direct *in vivo* analysis of elongation on chromatin^40^. In this assay, the amount of nascent mRNA synthesized in the cells over a promoter distal G-less cassette is compared to the amount of mRNA synthesized over a promoter proximal G-less cassette, the two cassettes being separated by a long, G-rich sequence that is refractory to elongation (Fig. 6a). RNase T1 digestion of total cellular RNA degrades all G-containing sequences leaving the two G-less cassettes intact that can be visualized and measured after polyacrylamide gel electrophoresis. In our hands, transcription elongation efficiency of the second cassette in the control *spt4Δ* strain, used originally to validate the method, was ~20% of the wild-type value, in good agreement with the published data (Fig. 6b and c). Notably, in *rad30Δ* cells, elongation efficiency was reduced to ~60% of the wild-type level. These observations are consistent with the results obtained from the RT-qPCR and luciferase assays shown in Fig. 5 and support a potential role of Polη in transcription elongation.

### Polη is enriched over the actively transcribed *GAL1* gene

Next we investigated whether Polη co-localized with transcriptionally active regions, as predicted by the above experiments. For this purpose we examined the enrichment of Myc-tagged Polη at the *GAL1* gene relative to an intergenic region, using chromatin immunoprecipitation (ChIP) experiments^41,42^. To avoid detecting enrichment due to ongoing replication, cells were arrested in the G1 phase of the cell cycle (Fig. S9). Our results show that whereas a small, 2 fold increase could be detected at the upstream activating sequence (UAS) when shifting cells from raffinose to galactose, the level of Polη enrichment increased 5–8 fold over the open reading frame (ORF) of the *GAL1* gene after transcription induction (Fig. 6d). In contrast, no increase could be observed over a non-transcribed intergenic region. As controls, chromatin immunoprecipitations were performed with or without galactose induction in Gcn5-Myc and Spt5-Myc expressing as well as in non-tagged strains. In these experiments, as expected, the transcriptional co-activator histone acetyltransferase Gcn5 increased preferentially at the UAS (Fig. S9b), whereas the elongation factor Spt5 exhibited high enrichment over the ORF (Fig. S9c). In the non-tagged control strain, non-specific enrichment could not be detected. Taken together, these data show preferential enrichment of Polη over the ORF of the active *GAL1* gene suggesting that Polη is specifically recruited to sites of active transcription.

### The catalytic activity of Polη is involved in its role in transcription

**Figure 7:**
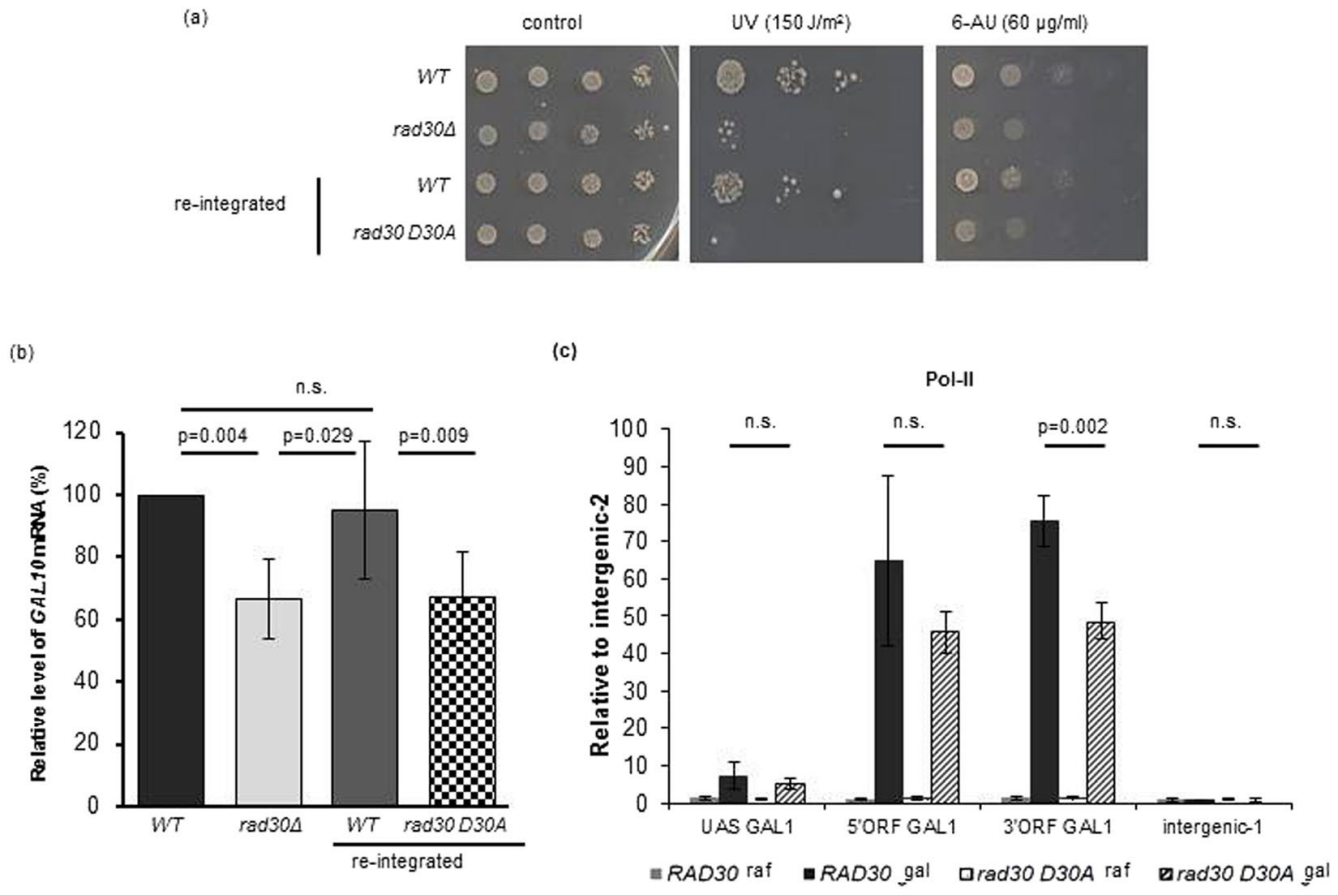
Catalytic inactivation of Polη causes similar defects as the deletion of the *RAD30* gene. (**a**) Strains carrying the Polη D30A mutant are sensitive to both UV and 6-AU. Ten fold serial dilutions of the indicated strains were spotted on media containing the indicated concentration of 6-AU, or irradiated with the indicated UV-dose after spotting. (**b**) Induced level of *GAL10* mRNA is decreased in the Polη D30A mutant, as measured by RT-qPCR. The mean value obtained for the wild-type strain was set to 100% and the values obtained with the other strains are shown relative to that. (**c**) Occupancy of PolII in the Polη-Myc and the D30A Polη-Myc mutant expressing strains was measured with ChIP using anti-Myc antibody as described in Fig. 6d. Mean and standard deviations based on at least 4 experiments are indicated, p-values were calculated by 2-tailed t-test,n.s.: no statistical difference.

The polymerase activity of Polη is essential for its known *in vivo* functions during DNA synthesis^43^. To address whether the polymerase activity of Polη was necessary for its new transcriptional role as well, we generated a strain expressing the catalytically inactive Polη D30A mutant from the genomic *RAD30* locus. This was achieved by integrating back the wild-type or a mutant *RAD30* copy coding for the D30A mutant protein into a *rad30Δ* strain. First, we verified by Western blot analysis that both the wild-type and mutant reintegrated genes expressed similar Polη levels (Fig. S10a). Next, we examined the sensitivity of the strains to different agents. As expected, reintegration of the wild-type sequence suppressed the UV and 6-AU sensitivities of the *rad30Δ* strain to the wild-type level confirming that both the enhanced UV and 6-AU sensitivities were indeed due to the lack of Polη (Fig. 7a). On the other hand, reintegration of the sequence coding for the D30A mutant protein rescued neither the UV nor the 6-AU sensitivity of the *rad30Δ* null mutant. Accordingly, the Polη D30A mutant negatively affected activation of *GAL10* and *GAL1* genes similarly to *rad30Δ*, whereas this was not the case for the reintegrated wild-type *RAD30* (Fig. 7b and Fig. S10b). Importantly, chromatin immunoprecipitation experiments showed that the association of RNA PolII CTD with the active *GAL1* gene was significantly reduced (p = 0.0015) at the 3′ end in the D30A mutant compared to wild type, consistent with a defect in transcription elongation in this strain (Fig. 7c). In summary, these results indicate that the catalytic activity of Polη is required for its role in transcription.

## Discussion

The results presented in this report uncover a specific RNA synthesis activity for the TLS DNA polymerase Polη. Furthermore, *in vivo* experiments establish a link between Polη and transcription.

The specificity of the novel RNA synthesis activity of Polη was assessed in steady-state kinetic experiments. This analysis revealed that Polη recognizes RNA as its substrate incorporating rNTPs into RNA an order of magnitude more efficiently than into DNA. Furthermore, Polη could carry out TT dimer and 8-oxoG bypass during RNA extension by preferentially incorporating the correct A or C residue opposite the damage, respectively. These observations are paralleled by *in vivo* experiments supporting a connection between Polη and transcription. By measuring mRNA levels from inducible and constitutive promoters, we showed that transcription was generally diminished in the absence of Polη. This defect was independent of the DNA synthetic role of Polη as it could be detected even when replication and repair synthesis were inhibited. Chromatin immunoprecipitation and *in vivo* transcription run-on assays demonstrated that Polη was enriched over the ORF of the active *GAL1* gene and affected transcription elongation. Taken together the *in vivo* results suggest a role for Polη in transcription thereby providing a possible setting for its specific RNA synthesis activity. In particular, the observation that the catalytically inactive protein causes similar defects in transcription *in vivo* as the lack of Polη suggests that the newly discovered specific RNA synthesis activity of Polη contributes to transcription. Probably the most obvious reason for Polη being associated with transcription could be to rescue stalled RNAPII, particularly by inserting ribonucleotides opposite DNA lesions, similar to its role in rescuing damage-stalled replication. Although *in vitro* damage bypass studies showed that RNAPII could bypass several small, non-distorting lesions, such as abasic sites, dihydrouracil and 8-oxoG, resulting in mutagenic transcripts^44–46^, still, bulky damages and CPDs induced a complete block to RNAPII. *In vivo* bypass of these lesions in nucleotide excision repair (NER) defective cells further suggested the existence of damage bypass mechanisms operating during transcription^47–53^. So far translesion RNA synthesis has been thought to be performed by RNAPII itself with the aid of elongation factors. Indeed, TFIIF, TFIIS, Elongin and CSB purified from HeLa cells were shown to help RNAPII to bypass certain oxidative DNA lesions^54^. Nevertheless, it remains elusive how RNAPII could bypass the high variety of lesions, and how it could overcome the blocks represented by CPDs and bulky damages. Based on our results, Polη could be a new candidate to help RNAPII to bypass DNA damage sites. However, our kinetic experiments demonstrated that Polη inserted dNTPs and rNTPs into RNA with similar efficiencies at intracellular nucleotide concentrations. Furthermore, though Polη could carry out error-free bypass of a TT dimer and 8-oxoG during RNA extension, damage bypass was an order of magnitude more efficient with dNTPs at intracellular nucleotide concentrations. These results indicate that if Polη participated in RNA synthesis *in vivo*, it could insert both rNTPs and dNTPs on undamaged templates, and it would most probably insert dNTPs opposite DNA lesions. This would have severe consequences by leading to the accumulation of dNTPs in RNA causing miscoding, affecting RNA structure^55^, RNA-protein interactions^56^, and RNA packaging^57^. To resolve this problem we presume that cellular factors can modify the kinetics of the reactions. Indeed, the DNA replication factor PCNA together with the clamp loader RFC and the ssDNA binding protein RPA were shown to stimulate the DNA synthetic activity of Polη with an order of magnitude on undamaged templates, and with two orders of magnitude opposite an abasic residue^11,12^. To get access to the nascent RNA, Polη is likely to form interactions with members of the elongation machinery, and as with its interaction with replication factors, these interactions could modulate the activity and/or selectivity of Polη so that rNTP insertion would be preferred opposite to undamaged as well as damaged bases.

Our hypothesis introduces a new concept: transcriptional DNA lesion bypass mediated by polymerase switch. Polymerase switch during transcription has long been ruled out because of the need for specific promoter elements to start transcription. Nevertheless, RNAPII has been shown to be able to pause, move backwards at stall sites, and transcription could be reactivated from the stalled state proving the elongating complex to be much more flexible than previously assumed^58–60^. We presume that this flexibility could support the consecutive steps of polymerase exchange at transcription complexes stalled at DNA damage sites.

Our hypothetical model could explain puzzling earlier observations detecting mammalian Polη foci formation after UV treatment in cells where replication and repair synthesis were inhibited^61^. Polη foci formation occurred in chinese hamster ovary and in human cell lines arrested in the G1 phase of the cell cycle. It was independent of PCNA, and inactivation of NER did not influence Polη foci, either. Several studies demonstrated that transcription takes place at discrete foci in the nucleus called transcription factories^62–64^. We suggest that the transcriptional role of Polη might be conserved through evolution and that the observed foci formation of mammalian Polη could represent its recruitment to stalled transcription elongation complexes at transcription factories.

During the course of this work, a few studies have been published examining rNTP incorporation by yeast or human Polη. They showed that yeast Polη is very inefficient in extending a DNA primer with rNTPs^20^, whereas human Polη can extend a DNA primer with several rNTPs and even bypass DNA lesions such as 8-oxoG and TT dimers using ribonucleotides^65,66^. However, to our knowledge, the current study is the first showing specific RNA extension by a DNA polymerase and implicating this activity in transcription.

## Methods

### Yeast strains and plasmids

The wild-type strain BY4741 (*MATa, his3-Δ1, leu2, met15, ura3*) and its single deletion derivatives were obtained from the Euroscarf collection. Additional deletions were generated by gene replacement. Strains used in experiments involving synchronizing the cells in G1 phase were made *bar1Δ* to achieve complete and stable cell cycle arrest. For detection during chromatin immunoprecipitation, 9 copies of the Myc tag was fused to the C-terminus of the *RAD30, GCN5*, and *SPT5* genes at the genomic locus by applying a PCR-based method^67^. To generate the mutant Polη protein, site-specific mutagenesis was carried out by a PCR based method according to the “Quick Change Site Directed Mutagenesis” protocol (Stratagene, La Jolla, California). Reintegration of wild-type or mutant *RAD30* was done by transforming a linear DNA fragment containing the *RAD30* gene from −317 to 900 nucleotides after the stop codon, with the *HIS3* marker gene inserted 632 nucleotides downstream of the stop codon, into *rad30Δ* yeast cells. Genomic changes were confirmed by PCR and sequencing. The protease deficient yeast strain BJ5464 (*MATα*, *his3-Δ*200, *leu2-Δ*1, *trp1Δ*, *ura3-52, pep4::HIS3, prb1-Δ1.6 R, can1*) was used for protein overexpression (ATCC stock centre). pCYC-LacZ (GLRO-Long) was used for GLRO assays^40^. The pY25GAL1-GPD dual promoter plasmid (Turbobiotech, Chang Cun, China) was used to clone the Renilla and Firefly luciferase genes under the *GPD* and *GAL1* promoters, respectively. The luciferase genes with the respective promoters and terminators were further cloned into the centromeric plasmid YCplac33 to generate plasmid pID723 used in the luciferase assays. For protein purification, the wild-type and the D30A mutant Polη were overexpressed as N-terminal fusions with the *glutathione S-transferase* (GST) gene from pID206 and pID797, respectively (pBJ842 backbone)^68^.

### Polη purification

Wild-type and D30A mutant Polη were overexpressed in yeast as N-terminal fusions with GST and affinity purified on glutathione–Sepharose 4B beads (GE Healthcare) in a buffer containing 50 mM Tris/HCl pH 7.0, 50 mM KCl, 100 mM NaCl, 10% sucrose, 0.5 mM EDTA, 10 mM 2-mercaptoethanol and protease inhibitors. After washing the column three times with 10X volume of a buffer containing 100 mM Tris/HCl pH 7.5, 1 M NaCl, 0.01% NP40, 10% glycerol, and then two times with the same buffer but containing 100 mM NaCl, the GST-tag was removed in the last step of the purification by incubating the beads with PreScission protease in a buffer (50 mM Tris/HCl pH 7.5, 150 mM NaCl, 1 mM EDTA, 1 mM DTT, 0.01% Nonidet P-40, 10% glycerol) at 4 °C for 12 hours.

### Primer extension assays

Standard reactions (5 µl) contained 25 mM Tris/HCl pH 7.5, 5 mM MgCl_2_, 1 mM dithiothreitol, bovine serum albumin (100 µg/ml), 10% glycerol, and the specified amount of template and substrate. Reactions were initiated by the addition of wild-type or mutant Polη at the indicated concentrations, incubated at 30 °C and quenched by the addition of 10 µl loading buffer containing 95% formamide, 18 mM EDTA, 0.025% SDS, 0.025% bromophenol blue and 0.025% xylene cyanol. The reaction products were resolved on 10% polyacrylamide gels containing 8 M urea and analyzed with a Typhoon TRIO Phosphorimager (GE Healthcare). Oligonucleotides used in these experiments were purchased from Integrated DNA Technologies, San Jose, California, except for the 8-oxoG containing primer that was from Midland Certified Reagent Co. Midland, Texas, and the TT-dimer containing oligonucleotide was from Trilink Biotechnologies, San Diego, California. Oligonucleotide sequences and the structures of substrates are shown in Table 1. To facilitate detection, oligonucleotides labeled with the fluorophore indocarbocyanine (Cy3) at the 5′-ends were used as primers.

**Table 1:** Sequence and structure of substrates used in the *in vitro* primer extension assays.

Substrate	Sequence
S1	/5Cy3/CGCTACCTAGCCTGCCTCAAGAGTTGCTCG 3′-GCGATGGATCGGACGGAGTTCTCAACGAGC**A**CAGGCTTACGCTCAGGTCG-5′
S2	/5Cy3/CGCTACCTAGCCTGCCTCAAGAGTTGCTCG 3′-GCGATGGATCGGACGGAGTTCTCAACGAGC**T**CAGGCTTACGCTCAGGTCG-5′
S3	/5Cy3/CGCTACCTAGCCTGCCTCAAGAGTTGCTCG 3′-GCGATGGATCGGACGGAGTTCTCAACGAGC**G**CAGGCTTACGCTCAGGTCG-5′
S4	/5Cy3/CGCTACCTAGCCTGCCTCAAGAGTTGCTCG 3′-GCGATGGATCGGACGGAGTTCTCAACGAGC**C**CAGGCTTACGCTCAGGTCG-5′
S5	/5Cy3/CGCUACCUAGCCUGCCUCAAGAGUUGCUCG 3′-GCGATGGATCGGACGGAGTTCTCAACGAGC**A**CAGGCTTACGCTCAGGTCG-5′
S6	/5Cy3/CGCUACCUAGCCUGCCUCAAGAGUUGCUCG 3′-GCGATGGATCGGACGGAGTTCTCAACGAGC**T**CAGGCTTACGCTCAGGTCG-5′
S7	/5Cy3/CGCUACCUAGCCUGCCUCAAGAGUUGCUCG 3′-GCGATGGATCGGACGGAGTTCTCAACGAGC**G**CAGGCTTACGCTCAGGTCG-5′
S8	/5Cy3/CGCUACCUAGCCUGCCUCAAGAGUUGCUCG 3′-GCGATGGATCGGACGGAGTTCTCAACGAGC**C**CAGGCTTACGCTCAGGTCG-5′
S9	/5Cy3/CGACGATGCTCCGGTACTCCAGTGTAGGCAT 3′-AAAGGGTCAGTGCTGCTACGAGGCCATGAGGTCACATCCGTA**G**AATGCTTAAGAA CTCCGTCCGTACCATCGA-5′
S10	/5Cy3/CGACGATGCTCCGGTACTCCAGTGTAGGCAT 3′-AAAGGGTCAGTGCTGCTACGAGGCCATGAGGTCACATCCGTA**°G**AATGCTTAAGAA CTCCGTCCGTACCATCGA-5′
S11	/5Cy3/CGACGAUGCUCCGGUACUCCAGUGUAGGCAU 3′-CAAAAGGGTCAGTGCTGCTACGAGGCCATGAGGTCACATCCGTA**G**AATGCTTAAGAA CTCCGTCCGTACCATCGA-5′
S12	/5Cy3/CGACGAUGCUCCGGUACUCCAGUGUAGGCAU 3′-CAAAAGGGTCAGTGCTGCTACGAGGCCATGAGGTCACATCCGTA**°G**AATGCTTAAGAA CTCCGTCCGTACCATCGA-5′
S13	/5Cy3/CGTATTCGCGCGC 3′-CGAATGGCGGTGCG**T**TGCGCGCGAATACG-5′
S14	/5Cy3/CGTATTCGCGCGC 3′-CGAATGGCGGTGCG**T^T**GCGCGCGAATACG-5′
S15	/5Cy3/CGUAUUCGCGCGC 3′-CGAATGGCGGTGCG**T**TGCGCGCGAATACG-5′
S16	/5Cy3/CGUAUUCGCGCGC 3′-CGAATGGCGGTGCG**T^T**GCGCGCGAATACG-5′

RNA primers are underlined. The Cy3 label at the 5′ end of primers is indicated. The first templating nucleotides are in bold.

### Determination of steady-state kinetic parameters

For steady-state kinetics of RNA and DNA primer extensions with rNTPs on undamaged templates, Polη (1 nM) was incubated with 20 nM of the DNA:DNA (S1-4)or DNA:RNA (S5-8) templates in standard buffer (as above). For dNTP insertion into RNA, 1 nM Polη was incubated with 24 nM template in standard buffer. Reactions were initiated by adding the corresponding single rNTP (varied from 0.25 to 4 mM) or dNTP (25-1000 µM), and incubated at 30 °C from 30 sec to 60 min. The intensity of the gel bands corresponding to the substrate and the product were quantitated with Typhoon TRIO Phosphorimager (GE Healthcare) using ImageQuant TL software (GE Healthcare) and the observed rates of nucleotide incorporation were plotted as a function of rNTP concentration. The data were fit by nonlinear regression using SigmaPlot program (version 12.5 Systat Software, San Jose, CA) to the Michaelis-Menten equation describing a hyperbola, *v* = (*V*_max_ X [rNTP]/(*K*_*m*_ + [rNTP]). The *k*_*cat*_ and *K*_*m*_ steady-state parameters were obtained from the fit and were used to calculate the efficiency of rNTP insertion into RNA versus DNA by using the following equation: *f*_*ext*_ = (*k*_*cat*_/*K*_*m*_)_RNA_/(*k*_*cat*_/*K*_*m*_)_DNA,_ and dNTP versus rNTP insertion into RNA according to the formula *f*_*rel*_ = (k_cat1_/K_m1_) * [dNTP]**/**(k_cat2_/K_m2_) * [rNTP]^69^.

### Steady-state kinetic assay of DNA lesion bypass

For kinetic analysis of 8-oxoG bypass, 1 nM Polη was incubated with 8 nM template (S12) in standard buffer. Reactions were initiated by adding rCTP (0.25 to 4 mM) or dCTP (25–1000 µM), and incubated at 30 °C for 30 min and 1 min, respectively. In case of TT dimer, 1 nM Polη was incubated with 16 nM template (S16) in standard buffer. Reactions were initiated by adding rATP (0.25 to 4 mM) or dATP (25–1000 µM), and incubated at 30 °C for 60 min and 2 min, respectively. Reactions were visualized on 12% polyacrylamide gels containing 8 M urea and quantitated as above.

### Sensitivity Assays

For 6-AU sensitivity assays, strains were transfected with YCplac33 (URA3 expressing plasmid) and cultures were grown overnight in synthetic complete (SC) media lacking uracil (-ura). From these starter cultures, 10X serial dilutions were spotted on SC-ura plates containing the respective amounts of 6-AU. MPA sensitivity was assayed similarly, but cells were grown in and spotted on SC media containing the indicated amount of MPA. Plates were incubated at 30 °C for 4–5 days. For UV sensitivity assays, 10X serial dilutions of overnight cultures grown in YPD (yeast-peptone-dextrose) medium were spotted on YPD plates, irradiated with the respective UV doses and incubated in the dark at 30 °C for 2–3 days.

### Luciferase Assays

Strains transformed with pID723 and grown in SC-ura medium were used to measure luciferase activity using the dual luciferase reporter assay system (Promega Corporation, Madison, Wisconsin). Firefly luciferase expression was induced by addition of 2% galactose to cultures at a density of A_600_:0.7 and after 1 h cells were harvested. To measure the constitutive expression of renilla luciferase, logarithmically growing cells at A_600_:0.7 were counted before measurements using a Bürker chamber, and activity was normalized to cell number. Luciferase measurements were carried out as described using a Fluoroskan Ascent FL microplate fluorometer and luminometer (Thermo Fischer Scientific Inc., Waltham, Massachusetts)^70^.

### Analysis of mRNA levels by RT-qPCR

For measurement of *IMD2*, *UBC6* and *TRP3* mRNA levels, yeast strains transformed with YCplac33 were grown in SC-ura medium at 30 °C with vigorous shaking. At A_600_:0.5, 6-AU was added to a final concentration of 70 µg/ml. After 2 h cells were collected and quickly frozen at −80 °C. For induction of *GAL* genes, yeast strains were grown in SC medium containing lactate as the sole carbon source (SCL). At A_600_:0.5, MPA was added to a final concentration of 70 µg/ml. After 2 h, galactose was added to a final concentration of 2% to induce *GAL* gene expression. 1 h after induction cells were collected and quickly frozen at −80 °C. For synchronization in the G1 phase of the cell cycle, exponentially growing strains carrying deletion of the *BAR1* gene were synchronized at A_600_:0.4 in SCL by adding alpha mating factor (Sigma-Aldrich, St. Louis, MO, USA) to a final concentration of 50 ng/ml. Synchronization was checked microscopically. After 3 h MPA was added to a final concentration of 70 µg/ml, as well as more alpha factor to keep the cells in G1 phase. After 2 h with MPA, galactose was added to a final concentration of 2% and after another hour of incubation at 30 °C cells were pelleted and quickly frozen to −80 °C. Total RNA was purified using TRIzol Plus kit (Life Technologies, Carlsbad, California) according to the manufacturer’s protocol, except cells were disrupted with glass beads. On-column DNase treatment was performed for 20 minutes using PureLink DNase. Reverse transcription of 0.5 μg RNA was performed using oligo-dT primer and Revert Aid first strand cDNA synthesis kit (Thermo Fischer Scientific). Real-time qPCR was performed with SYBR-Green detection method on Light Cycler 480 (Hoffmann-La Roche, Basel, Switzerland) with the primers summarized in Table S1. The *SED1* gene, whose mRNA level does not change significantly upon 6-AU or MPA treatment, or upon deleting *RAD30*, was used for normalization^71^.

### G-less based run-on assay (GLRO)

GLRO assays were carried out as previously described^40,72^. Briefly, the wild-type and mutant strains harboring the GLRO-long plasmid pCYC-LacZ were grown to an A_600_:0.5 in SC-leu at 30 °C. Cells were permeabilized with 0.5% sarkosyl for 20 min on ice. Pelleted cells were resuspended in 71 μl of ice-cold transcription mix (42.25 mM Tris/HCl pH 7.7, 422.5 mM KCl, 67.6 mM MgCl_2_, 1.13 mM ATP, 1.13 mM CTP, and 4.225 mM dithiothreitol). Labeling of nascent transcripts was initiated by the addition of 50 μCi of [α−32P]UTP (3,000 Ci/mmol), and samples were incubated for 5 min at 27 °C. “Chase” was performed with the addition of 10 μl of 25 mM UTP–0.25 mM GTP for 10 min at 27 °C. Reactions were stopped by addition of 900 μl of ice-cold AE buffer (50 mM sodium acetate, 10 mM EDTA pH 5.0). Total RNA was isolated using TRIzol (Life Technologies) and digested with RNaseT1, which only leaves G-less cassettes intact, for 2 h at 37 °C. After proteinase K treatment (Thermo Scientific), the remaining RNA was precipitated with ethanol as described, resuspended in formamide gel loading buffer (Life Technologies) and run on a 6% denaturing urea-acrylamide gel. Dried gels were analyzed with Typhoon TRIO Phosphorimager (GE Healthcare, Little Chalfont, UK) using ImageQuant TL software (GE Healthcare) as described^40^.

### Chromatin immunoprecipitation (ChIP)

For ChIP experiments, cells were exponentially grown at 30 °C in SC + 2% raffinose medium to A_600_:0.6–0.8 and arrested in G1 during 4 h with alpha-factor (20 ng/ml). Synchronization efficiency was measured by FACS. To induce *GAL1* gene expression 2% galactose was added to G1-arrested cells and cultures were grown for 1 h at 30 °C. Non-induced (2% raffinose) or induced (2% galactose) yeast cultures were cross-linked with 1% formaldehyde during 15 minutes and neutralized with 250 mM glycine during 5 min at room temperature followed by 10 min on ice. Cultures were pelleted and washed twice with cold PBS buffer. All subsequent procedures were done at 4 °C unless otherwise stated. Pellets were resuspended in FA buffer (50 mM HEPES-KOH pH 7.5, 140 mM NaCl, 1 mM EDTA, 1% Triton X-100, 0.1% sodium deoxycholate, protease inhibitor cocktail (Roche)) and cells were disrupted with glass beads using a MagNA Lyser (4 times, 6000 rpm). Recovered lysates were sonicated to obtain chromatin fragments with an average size of 250–500 nt and centrifuged for 15 min at 18000 g. The protein concentration of the supernatant was measured by Bradford and 1.5 mg of total cell extract was incubated overnight with antibodies (anti-Myc 9E10 or anti-CTD PolII 8WG16 (Abcam)) and an additional 3 h with Dynabeads® Protein G (Thermo Fisher Scientific). After immunoprecipitation, beads were washed once with FA buffer, twice with FA buffer + 500 mM NaCl, twice with buffer III (10 mM Tris-HCl, pH 8.0, 1 mM EDTA, 250 mM LiCl, 1% NP-40, 1% sodium deoxycholate) and once with TE buffer (10 mM Tris-HCl pH 8.0, 1 mM EDTA). The immunoprecipitated material was eluted from the beads by two sequential incubations in 100 µl of buffer B (50 mM Tris-HCl, pH 7.5, 1% SDS, 10 mM EDTA) at 65 °C during 8 min. Proteinase K (Roth) was added to the eluted material and to input (10% of total IP volume set aside before antibody addition) to a final concentration 0.75 mg/ml and incubated at 42 °C for 2 h. De-crosslinking was done at 65 °C for 15 h followed by DNA purification by Wizard® SV Gel and PCR Clean-Up System (Promega). Quantification of total or precipitated DNA was done by RT-qPCR using SYBR® Green PCR Master Mix kit (Applied Biosystems) and primers listed in Table S2. Percentage of input for different regions was normalized to intergenic region 2 on the right arm of chromosome IV^42^.

### Western blot analysis

For checking the expression level of Polη, 50 ml yeast cultures grown in YPD were harvested at A_600_: ~1.0. Whole cell extracts were prepared by a glass-bead lysis method in 1xPBS (137 mM NaCl, 2.7 mM KCl, 10 mM Na_2_HPO_4_, 10 mM KH_2_PO_4_. 1 mM EDTA, 10% glycerol) with protease inhibitors. Cell lysates were quantified by Bradford. Equal amounts of whole cell lysates were separated by SDS-PAGE and analyzed by Western blotting using anti-Rad30 (sc-11868 Santa Cruz) and anti-PGK (Invitrogen A6457) primary antibodies, and anti-goat (sc-2020 Santa Cruz) and anti-mouse (Thermo Scientific 31430) secondary antibodies.

### Statistical analysis

Student’s t-test using Excel (Microsoft, Redmond, WA, USA) was applied to compare separate groups. p-values of < 0.05 were considered statistically significant.

### Data availability

All data generated or analyzed during this study are included in this published article (and its Supplementary Information files).

## Electronic supplementary material

Supplementary information
