# Supplementary material for: Translesion synthesis DNA polymerase η exhibits a specific RNA extension activity and a transcription-associated function
Source: Sci Rep. 2017 Oct 12;7:13055. doi: 10.1038/s41598-017-12915-1 (PMC5638924; doi:10.1038/s41598-017-12915-1)
Supplement: Supplementary file 1 — Supplementary information [file 41598_2017_12915_MOESM1_ESM.doc]

**Supplementary information**

**Translesion synthesis DNA polymerase η exhibits a specific RNA extension activity and a transcription-associated function**

Vamsi K. Gali^1,3¶^, Eva Balint^1¶^, Nataliia Serbyn^2^, Orsolya Frittmann^1^, Francoise Stutz^2^ and Ildiko Unk^1*^

^1^The Institute of Genetics, Biological Research Centre, Hungarian Academy of Sciences, Szeged, H-6726, Hungary

^2^Department of Cell Biology, iGE3, University of Geneva, 1211 Geneva, Switzerland

^3^Present address: Institute of Medical Sciences Foresterhill, University of Aberdeen, Aberdeen, United Kingdom

^¶^These authors contributed equally

^*^Corresponding author: unk.ildiko@brc.mta.hu


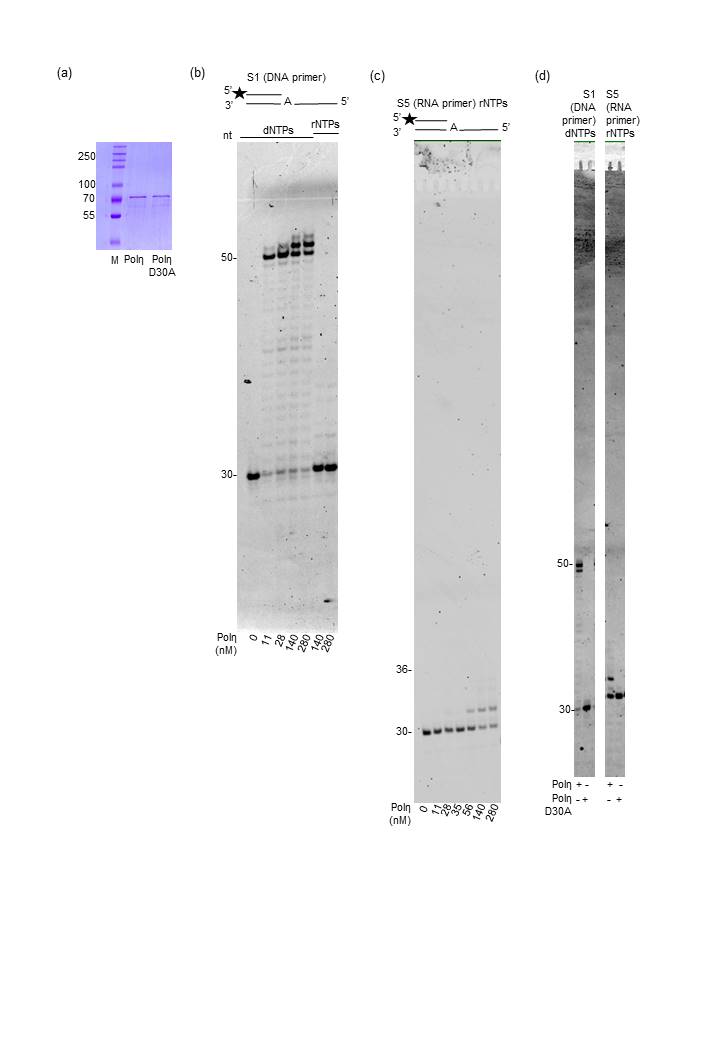


**Supplementary Figure S1**

Polη can catalyze DNA and RNA extension with rNTPs. Full-length images of pictures shown in **Fig. 1**.

(a)

Substrate S2 S3 S4 S1


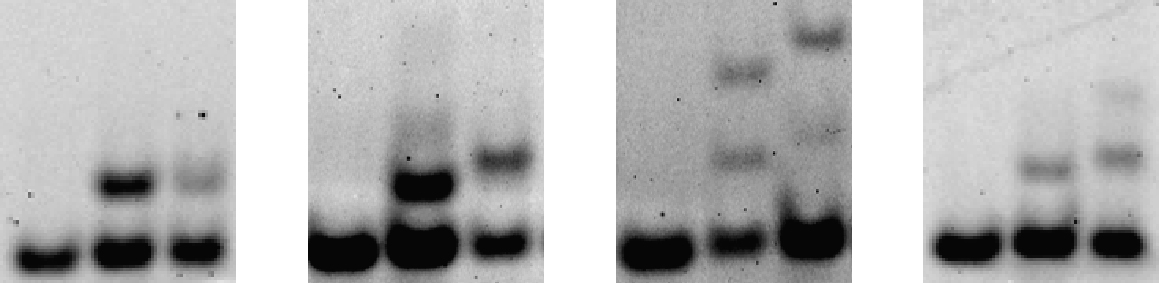


33-

31-

32-

dNTP/rNTP - dA rA - dC rC - dG rG - dT rU

(b)

Substrate S6 S7 S8 S5

31-

33-

32-

rNTP/dNTP - rA dA - rC dC - rG dG - rU dT

**Supplementary Figure S2**

Comparison of the electrophoretic mobility of (a) DNA primers, or (b) RNA primers extended with deoxyribonucleotides or ribonucleotides. Reactions were carried out with the indicated substrates incubated with Polη in the presence of a single dNTP or rNTP, as indicated. Reaction products were resolved in a 10% polyacrylamide gel containing 8 M urea.

**
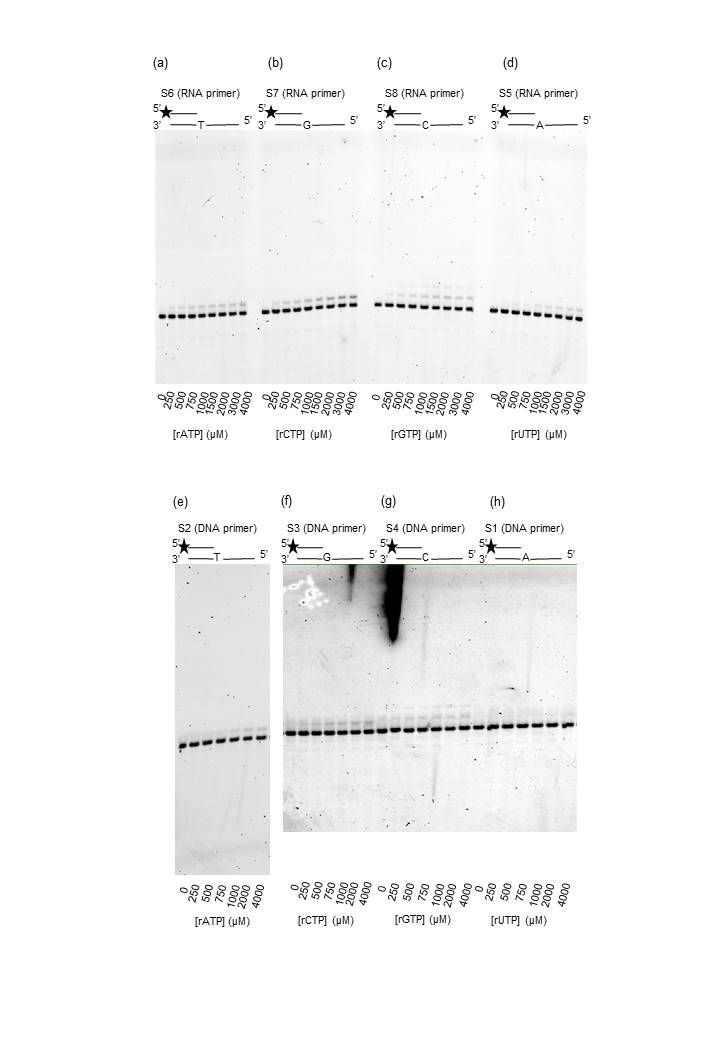
**

**Supplementary Figure S3**

Steady-state kinetic analysis of RNA and DNA primer extensions with rNTPs by Polη. Full-length images of pictures shown in **Fig. 2**.

**
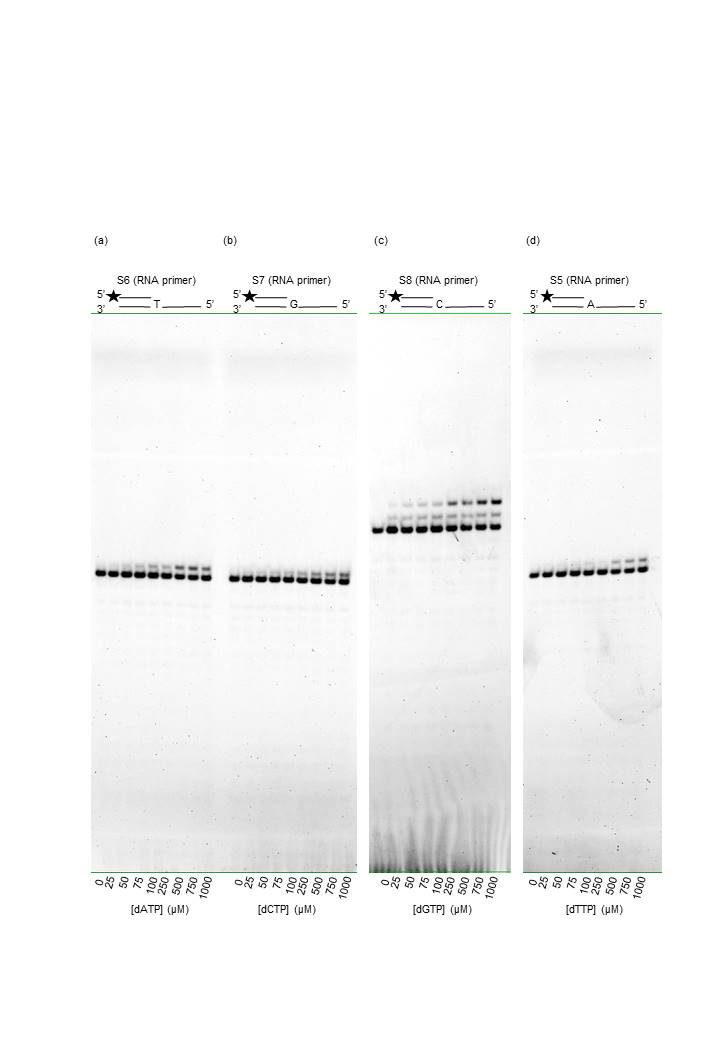
**

**Supplementary Figure S4**

Steady-state kinetic analysis of RNA extension with dNTPs by Polη. Full-length images of pictures shown in **Fig. 3**.

**
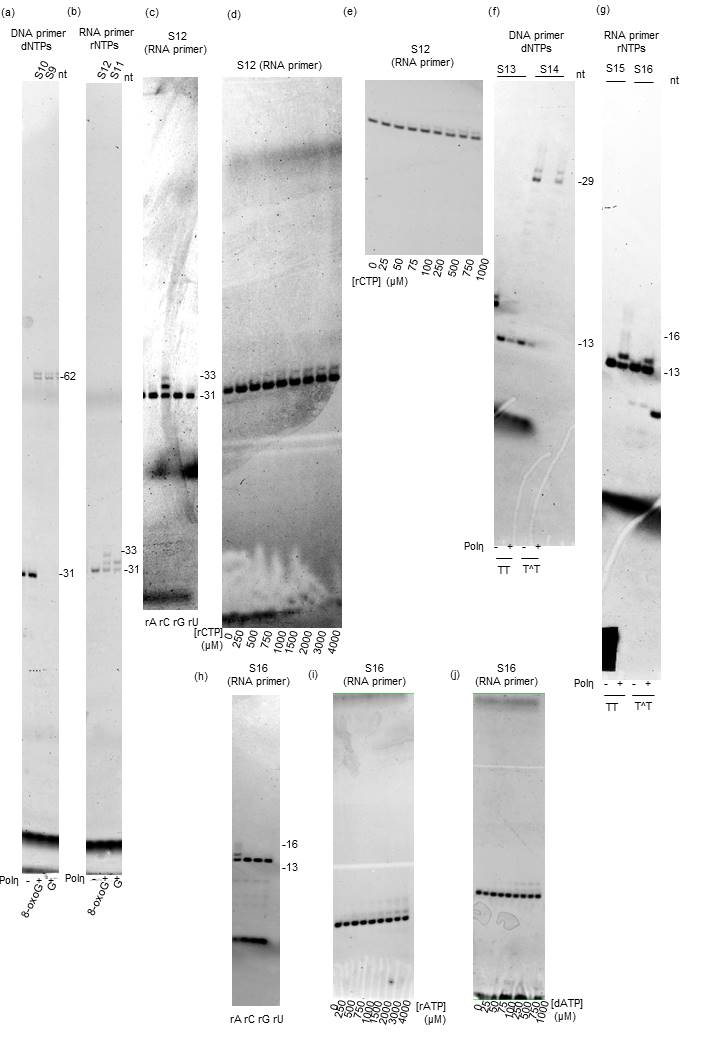
**

**Supplementary Figure S5**

Polη can carry out error-free bypass of 8-oxoG and TT dimer during RNA extension. Full-length images of pictures shown in **Fig. 4.**

(a) S12 (b) S12

(c) S16 (d) S16

**Supplementary Figure S6**

Steady-state kinetic analysis of 8-oxoG and TT dimer bypass during RNA primer extension by Polη. Insertion of (**a**) rCTP and (**b**) dCTP opposite an 8-oxoG. (**c**) rATP and (**d**) dATP insertion opposite a TT dimer. Reactions containing Polη (1 nM) and the indicated templates (8 nM) (a,b), or (16 nM) (c,d) were incubated in the presence of increasing concentrations of the single incoming rNTP or dNTP, as indicated. The quenched samples were analyzed by denaturing polyacrylamide gel electrophoresis, and quantified as described (see Materials and Methods). The observed rate of incorporation is plotted as a function of nucleotide concentration. The data were fit to the Michaelis-Menten equation (see Materials and Methods).

**(a)**

n.s.

n.s.

n.s.


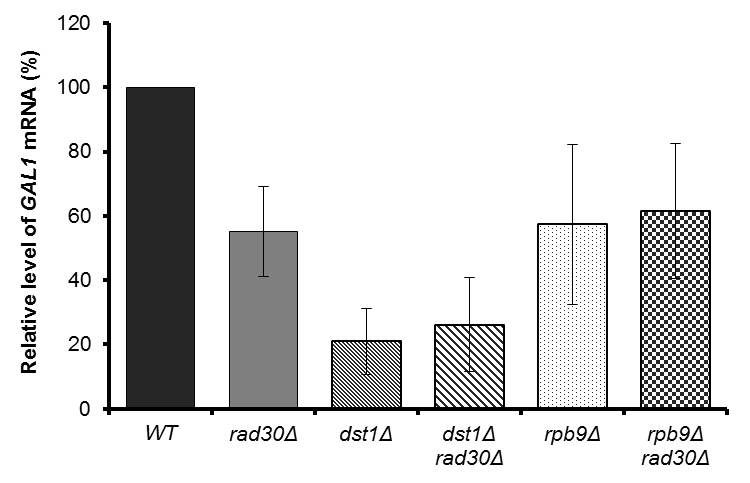


p=0.0002

p=0.0005

(b)


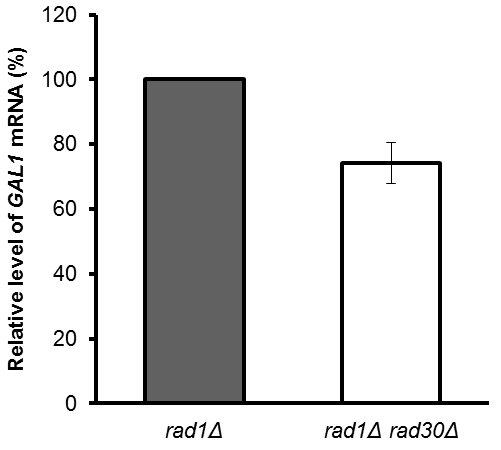


p=0.004

**Supplementary Figure S7**

Induced level of *GAL1* mRNA measured by RT-qPCR in the various strains. (**a**) *GAL1* mRNA levels were measured in logarithmically growing, non-synchronised, and (**b**) G1-arrested cultures: The values obtained for the wild-type (a), or the *bar1Δ* (b) strains were set to 100% and the values obtained with the other deletion strains are shown relative to that. mRNA levels was normalized to *SED1*. Experiments were repeated three times. Mean and standard deviations and p-values representing the significance of difference are also shown, ns: no statistical difference.


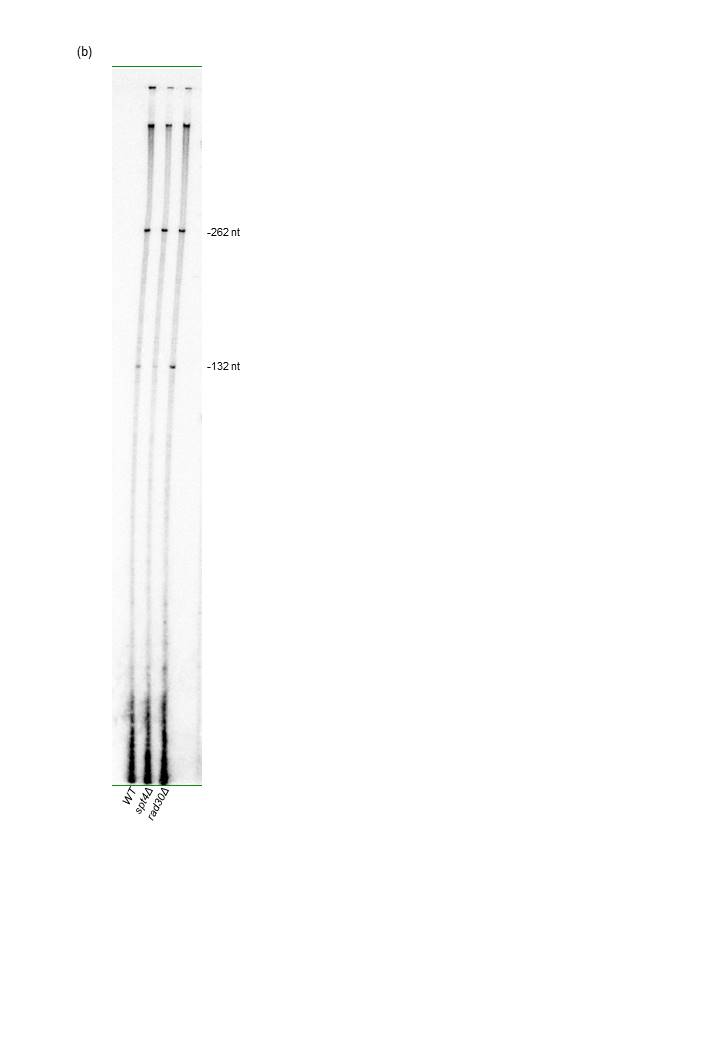


**Supplementary Figure S8**

Polη affects elongation *in vivo.* Full-length image of the picture shown in **Fig. 6b**.

**Supplementary Figure S9**

p=0.02 p=0.002 p=1*10^-5^

p=0.04 p=0.002 p=0.005

(a)

(b)

(c)

Control experiments for ChIP analysis. (**a**) Monitoring cell cycle stages during ChIP experiments. Representative fluorescence activated cell sorting (FACS) analyses of the Polη-Myc strain grown in SC + 2% raffinose before the addition of alpha-factor (left panel), after 4 hours synchronisation with alpha-factor (50ng/ml) (middle panel), and after 1 additional hour induction with 2% galactose (right panel). (**b**) Enrichment of Gcn5 and (**c**) Spt5 on the induced *GAL1* gene. Chromatin immunoprecipitation was performed on G1-arrested Gcn5-Myc and Spt5-Myc expressing strains in uninduced (raf) or induced (gal) condition using anti-Myc antibody or for control, ChIP was also performed with an untagged strain (no tag). Precipitated genomic regions were quantitated by qPCR using the primer pairs indicated below the graphs. Percentage of input at indicated regions was normalized to intergenic region 2. Experiments were repeated three times. Mean and standard deviations and P-values representing the significance of difference are also shown, n.s.: no statistical difference.

**
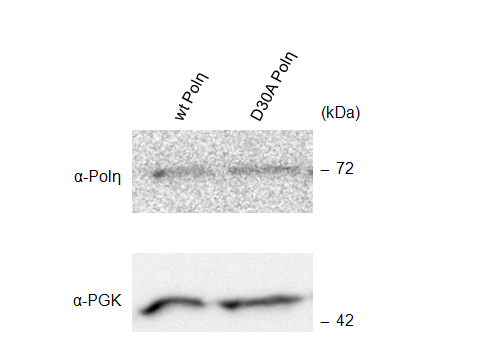
**

(b)

n.s.

p=0.02 p=0.04 p=0.03


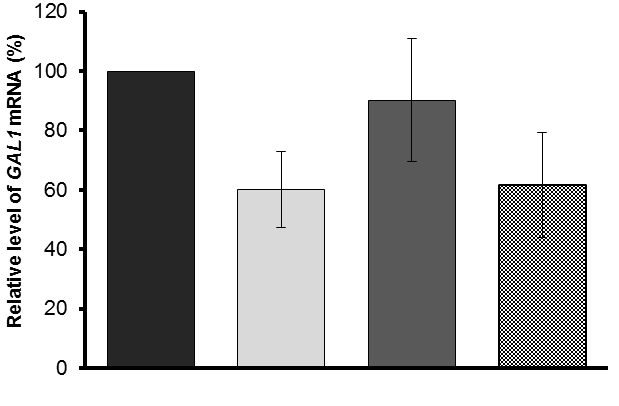


*

*

*

*WT rad30Δ WT rad30 D30A*

reintegrated

**Supplementary Figure S10**

Examination of the *RAD30* and *rad30 D30A* re-integrated strains. (**a**) The steady-state protein levels of the wild-type and the D30A mutant Polη expressed from the genes re-integrated into the genomic *RAD30* locus are similar. Wild-type or D30A mutant Polη was visualized in whole cell extracts prepared from 50 ml of logarithmically growing yeast cultures. 1 mg of total protein was separated on a 10% SDS-acrylamide gel and analyzed by Western blot using Polη-specific antibody. PGK served as a loading control. (**b**) Induced level of *GAL1* mRNA measured by RT-qPCR in the *RAD30* re-integrated strains. The values obtained for the wild-type strain were set to 100% and the values obtained with the deletion strains are shown relative to that. mRNA levels were normalized to *SED1*. Experiments were repeated three times. Standard deviations and p-values representing the significance of difference are also shown, ns: no statistical difference.

| **Gene name** | **Primer name** | **Primer sequence 5’ > 3’** | **Tm**  **(** ºC) | **Genomic position** | **Concentration in qPCR**  **(nM)** | **Efficiency** |
| --- | --- | --- | --- | --- | --- | --- |
| *GAL1* | GAL1 forward | GCTGCCTCTGTTTGCGGTGA | 60.8 | NC_001134.8 279675 .. 279694 | 200 | 2.018 |
|  | GAL1 reverse | AGTTGGTTGGGGCGGTTTCAA | 60.0 | 279840 .. 279820 |  |  |
| *GAL10* | GAL10forward | CTGCTGGTGAAGCGACGCCA | 63.0 | NC_001134.8 276779 .. 276760 | 200 | 2.042 |
|  | GAL10reverse | TGGGGCCTAAGACCGTTGGCT | 63.5 | 276573 .. 276593 |  |  |
| *IMD2* | IMD2 forward | GCATCTACCTCCCGTTACTTTTCCG | 59.2 | NC_001140.6 555692 .. 555716 | 200 | 1.989 |
|  | IMD2 reverse | TTTGTCAACGACAGCACCGGAG | 59.9 | 555769 .. 555748 |  |  |
| *SED1* | SED1 forward | GCGAAGCCCCTGAGTCTTCTGTC | 61.7 | NC_001136.10 601580 .. 601602 | 400 | 2.030 |
|  | SED1 reverse | ACTGGGACGACTGTGGAGACGG | 62.9 | 601703 .. 601682 |  |  |
| *TRP3* | TRP3 forward | GCCGCCTTGAATCCCGACACA | 60.0 | NC_001143.9  38000 37980 | 200 | 2.094 |
|  | TRP3 reverse | ACCGTGGACAATCTCACCAGCG | 59.0 | 37809 .. 37830 |  |  |
| *UBC6* | UBC6 forward | GGATGAGGGGGATGCGGCAAA | 62.5 | NC_001137.3 360071 .. 360091 | 200 | 1.917 |
|  | UBC6 reverse | ACGCTTGTTCAGCGCGTATTCTGT | 61.3 | 360198 .. 360175 |  |  |

**Supplementary Table 1**

Parameters of oligonucleotides used in the real time RT-qPCR experiments. Files containing further data regarding the compliance with MIQE (Minimum Information for Publication of Quantitative Real-Time PCR Experiments) are available from the investigators.

| **Region name** | **Primer name** | **Primer sequence 5’ > 3’** |
| --- | --- | --- |
| *GAL1* UAS | OFS1057 | TCCGACGGAAGACTCTCCTCC |
|  | OFS1058 | ATCTTTATTGTTCGGAGCAGTGCG |
| *GAL1* 5’ORF | OFS1707 | CTAAAGAAACTTGCACCGGAAAGG |
|  | OFS1708 | GCTAAAGCAACGGCACAAATGA |
| *GAL1* 3’ORF | OFS2782 | CATATGGTTCCCGTTTGACC |
|  | OFS2783 | TGGCAAGGGCTTCTTTTACC |
| Intergenic-1 Chr V | OFS2788 | tgttcctttaagaggtgatggtga |
|  | OFS2789 | gtgcgcagtacttgtgaaaacc |
| Intergenic-2 Chr IV | OFS710 | CGCATTACCAGACGGAGATGT |
|  | OFS711 | CAAGCAAGCCTTGTGCATAAGA |

**Supplementary Table 2**

Sequence of oligonucleotides used for qPCR after chromatin immunoprecipitation.
